# Influence of the Accuracy of Angiography-Based Reconstructions on Velocity and Wall Shear Stress Computations in Coronary Bifurcations: A Phantom Study

**DOI:** 10.1371/journal.pone.0145114

**Published:** 2015-12-21

**Authors:** Jelle T. C. Schrauwen, Antonios Karanasos, Nienke S. van Ditzhuijzen, Jean-Paul Aben, Antonius F. W. van der Steen, Jolanda J. Wentzel, Frank J. H. Gijsen

**Affiliations:** 1 Department of Biomedical Engineering, Thoraxcenter, Erasmus Medical Center, Rotterdam, the Netherlands; 2 Department of Cardiology, Thoraxcenter, Erasmus Medical Center, Rotterdam, the Netherlands; 3 Pie Medical Imaging, Maastricht, the Netherlands; 4 Department of Imaging Physics, Delft University of Technology, Delft, the Netherlands; University of Washington, UNITED STATES

## Abstract

**Introduction:**

Wall shear stress (WSS) plays a key role in the onset and progression of atherosclerosis in human coronary arteries. Especially sites with low and oscillating WSS near bifurcations have a higher propensity to develop atherosclerosis. WSS computations in coronary bifurcations can be performed in angiography-based 3D reconstructions. It is essential to evaluate how reconstruction errors influence WSS computations in mildly-diseased coronary bifurcations. In mildly-diseased lesions WSS could potentially provide more insight in plaque progression.

**Materials Methods:**

Four Plexiglas phantom models of coronary bifurcations were imaged with bi-plane angiography. The lumens were segmented by two clinically experienced readers. Based on the segmentations 3D models were generated. This resulted in three models per phantom: one gold-standard from the phantom model itself, and one from each reader. Steady-state and transient simulations were performed with computational fluid dynamics to compute the WSS. A similarity index and a noninferiority test were used to compare the WSS in the phantoms and their reconstructions. The margin for this test was based on the resolution constraints of angiography.

**Results:**

The reconstruction errors were similar to previously reported data; in seven out of eight reconstructions less than 0.10 mm. WSS in the regions proximal and far distal of the stenosis showed a good agreement. However, the low WSS areas directly distal of the stenosis showed some disagreement between the phantoms and the readers. This was due to small deviations in the reconstruction of the stenosis that caused differences in the resulting jet, and consequently the size and location of the low WSS area.

**Discussion:**

This study showed that WSS can accurately be computed within angiography-based 3D reconstructions of coronary arteries with early stage atherosclerosis. Qualitatively, there was a good agreement between the phantoms and the readers. Quantitatively, the low WSS regions directly distal to the stenosis were sensitive to small reconstruction errors.

## Introduction

Atherosclerosis is a chronic disease of the arterial system which leads to plaque formation. Plaque formation is a focal phenomenon, characterized by the accumulation of (low-density) lipoproteins, inflammatory cells and extra-cellular matrix in the arterial wall [1]. At first this process leads to thickening and outward remodeling of the arterial wall, which can subsequently progress to growth of plaque into the lumen. Rupture of plaques located in coronary arteries trigger thrombotic events, which are the main cause of acute myocardial infarction [2]. Understanding plaque development, progression and rupture is therefore of great clinical importance.

In the earliest phase of the disease, plaque development is triggered in zones of low or oscillatory wall shear stress (WSS) [3]. Especially sites near bifurcations or with a high curvature are prone to develop plaques. The largest study focusing on the effect of WSS on the progression of atherosclerosis in human coronary arteries was published by Stone et al. [4]. They investigated the role of WSS on plaque growth in 824 coronary arteries. They found that low WSS was an independent predictor of increased plaque burden and luminal obstruction over a 6 month period. Identification of low WSS regions in a clinical setting can therefore help identifying arterial segments that are prone to harbor progressing plaques leading to lumen narrowing. The role of WSS in the final phase of the disease process–plaque rupture- is less well established. Recent studies however indicated that increased WSS is related to changes in plaque composition that enhance plaque vulnerability [5–7]. These preliminary data are supported by clinical observations that regions exposed to elevated WSS are associated with the location of plaque rupture [8–11]. In summary, low WSS is associated with plaque progression and clinical lumen narrowing, while high WSS is potentially important to identify plaque locations that are at increased risk for plaque rupture.

WSS in coronary arteries cannot directly be measured in-vivo, but it can be computed with computational fluid dynamics (CFD). In order to perform CFD the geometry of the lumen is required, together with the inflow and outflow boundary conditions and the properties of the blood. Previous studies used a combination of angiography and intra-vascular ultrasound (IVUS) to reconstruct coronary arteries [12–14]. IVUS enabled high resolution reconstructions of the lumen. Nonetheless, reconstruction techniques using IVUS are time-consuming and require an additional catheter. The preferred imaging technique during percutaneous coronary interventions is angiography, which visualizes the lumen of coronary arteries. Reconstructions of coronary arteries can be made in real-time based on bi-plane angiography [15,16]. It was previously shown that accurate reconstructions can be made based solely on angiography [17] and they can potentially serve as the basis for WSS computation during clinical interventions [18–20]. The 3D reconstructions are based on segmentations on the two 2D projections. One of the main sources of errors in the reconstructions arises from inaccuracies of the segmentations by readers, what in turn is related to the resolution constraints. These reconstruction errors consequently influence the outcome of CFD.

Other studies explored the influence of imaging procedures on CFD results in coronary arteries. Most of these studies did not include ground truth data [20–25]. The study of Wellnhofer et al. on 3D coronary reconstruction based on angiography does not have ground truth data either, but they compare coronary reconstructions based on segmentation from different readers, concluding that the average difference is small [26]. However, the standard deviations reported in that study are fairly large, potentially leading to large differences in WSS. Goubergrits et al. used phantoms to validate image-based reconstruction procedures on geometry and WSS patterns [27]. They found that although the differences between the outer surfaces of the geometries were not negligible, WSS patterns were not affected significantly due to reconstruction inaccuracies [19]. This study focused steady flow simulations in a single scaled up healthy left coronary bifurcation model without atherosclerotic disease. Furthermore, the influence of user input on the segmentation procedure was not investigated.

The aim of this study was to analyze the effect of reconstructions errors on computed velocity and WSS in mildly-diseased coronary bifurcations using phantom models [28]. Mildly-diseased coronary arteries were investigated because these sites are affected by atherosclerosis but are normally left untreated. WSS can potentially give insight in the progression to more advance lesions and rupture risk. Secondly bifurcated regions were chosen. Although they are more difficult to reconstruct in 3D than single vessel segments, they are also more interesting since they are predilection sites for the onset of atherosclerosis. The phantom models were imaged with bi-plane angiography and two clinically experienced readers segmented lumen on the images. Based on the segmentations 3D reconstructions were made which were used for CFD computations. The simulations were performed to assess steady WSS, time-averaged WSS (TAWSS) and the oscillatory shear index (OSI). Computations were first performed in the original design of the phantom models and the results served as the ground truth. The results were quantitatively compared to those from computations in the 3D reconstructions.

## Methods

### 2.1 Phantoms

**Fig 1:**
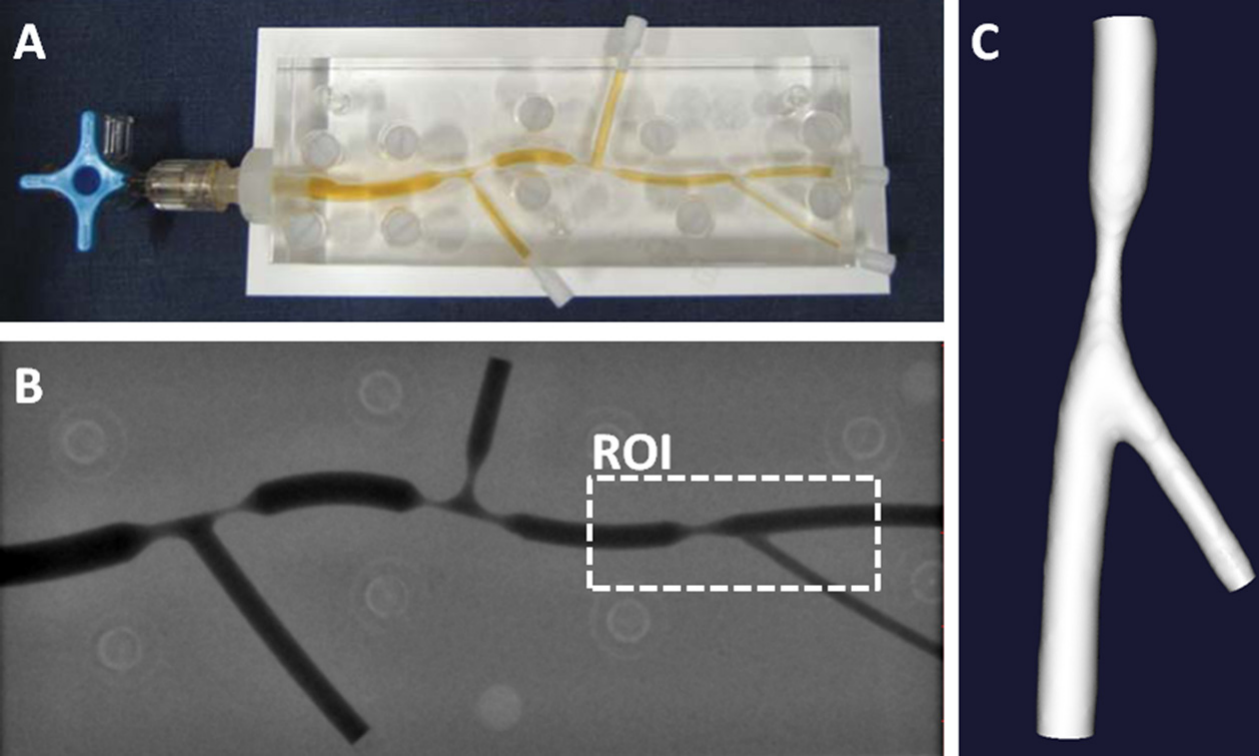
Overview of the 3D reconstruction steps of a phantom model with bi-plane angiography. **A:** Original phantom model filled with contrast agent. **B:** An angiography recording of the phantom. A region of interest (ROI) is indicated around a section that can be regarded as mildly stenosed. **C:** Final 3D reconstruction.

Plexiglass phantom models of diseased coronary bifurcations with concentric stenosis were previously created (Fig 1A) [28]. With computer-aided design three subsequent bifurcations were modeled, having at least one stenosis per bifurcation with the stenosis degree ranging from 40% to 80%. The phantoms had representative branch and stenosis lengths, diameters, and bifurcation angles. This study focused on flow phenomena in mildly diseased bifurcations. Four phantoms each had one bifurcation region that was representative for that status. These four regions of interest (ROI) consisted of a side branch and one stenosis in the main branch having an area stenosis of 60%. In three cases the stenosis was proximal to the side branch and in one case distal. The fabrication process of the phantom models was described in detail in Girasis et al. [28]. Briefly, the phantoms were designed with a computer-aided design program. The 3D models were used to instruct a computer controlled milling machine to mill these models in Perspex (Plexiglass). The accuracy of the machining process was reported to be within 10μm.

### 2.2 Imaging & Reconstruction

Digital angiograms were acquired with a bi-plane angiography (Axiom ArtisTM; Siemens, Forchheim, Germany). The phantoms were filled with contrast agent (100% Iodixanol 320, VisipaqueTM, GE Healthcare, Cork, Ireland) and imaged in a 20 cm field of view (Fig 1A and 1B) [17]. Of each phantom one set of bi-plane angiography images was selected. The angles were selected to achieve the best possible quantification of the side branch length and angle, as well as the degree of stenosis. The phantoms were acquired with a resolution ranging from 0.17 to 0.21 mm. The contrast-filled lumens in the 2D images were segmented by two clinically experienced readers blinded to the original geometry (AK, NvD). Based on the segmentations 3D models were generated with validated commercially available software: Cardiovascular Angiography Analysis System (CAAS v5.11, Pie Medical Imaging, Maastricht, the Netherlands [16,17]).

### 2.3 Mesh

First, the computer-aided designs of the phantoms were used to generate surface meshes of the ROI of the phantoms. Next, based on the 3D reconstructions of the two readers, surface meshes were generated using a newly developed module within the CAAS software package. A grid dependency study was performed prior to the final simulation. In subsequent steps the grid size was decreased until the pressure and wall shear stress did not differ more than 3% for each node. In particular attention was paid to the zone distal of the stenosis. This resulted in a typical cell size of 0.08 mm. Additionally a prism layer of 5 elements was added at the wall. The final mesh size was typically 3.10^6^ cells per mesh depending on the volume of the lumen (ICEM-CFD 14.5, Ansys Inc, Canonsburg, US). This resulted in three meshes per phantom: one gold-standard from the phantom model itself, and one mesh from each reader. From now on these models will be referred to as: phantom, reconstruction 1 and reconstruction 2. The radii of the reconstructions were acquired with VMTK (VMTK, Orbix, Bergamo, Italy) and compared to the radii of the phantom models. The mean Hausdorff distance between each point on the surface of phantoms and the surface of the reconstructions was computed using MeshLab (v1.3.3, open-source, http://meshlab.sourceforge.net/)

### 2.4 Computational fluid dynamics

Steady-state and transient CFD computations were performed with a commercially available finite volume solver (Fluent v14.5, Ansys Inc, Canonsburg, US). For the steady-state computations a Poiseuille profile was defined at the inlet of all models with a peak velocity of 15 cm/s, representative for normal physiological flow [29]. At the inlet this resulted in a Reynolds number of approximately 60 and at the stenosis approximately 200. For the transient simulations, typical flows curves of the coronary circulation were generated with a model developed by Bovendeerd et al. [30]. The flow cycles were defined to be 0.8 s. The flow curves were scaled such that the average flow corresponded to the flows in the steady-state simulations. Womersely profiles were prescribed at the inlet. Two full cardiac cycles were computed. Pilot studies showed that start-up effects were absent in the second cardiac cycle. Each flow cycle consisted of a 100 time steps. At the outlets of the bifurcations the flow ratio was defined using a diameter-based scaling law [31]. The wall was modeled as rigid. The blood was modeled as a non-Newtonian fluid using a Carreau model with the parameters taken from Cho and Kensey [32], and a density of 1060 kg/m3.

### 2.5 Wall Shear Stress analysis

The 3D WSS magnitude ($\left|\vec{\tau }\right|$) at the wall was extracted from the results of the steady-state computations. From the transient computations the 3D WSS vector ($\vec{\tau }$) was extracted, which was used to compute the time-averaged WSS (TAWSS):

$$TAWSS=\frac{1}{T}\int_{0}^{T}|\vec{\tau }|dt$$

In order to identify regions with oscillatory flow, the oscillatory shear index (OSI) was used [33]:

$$OSI=\frac{1}{2}\left[1-\frac{\left|\int_{0}^{T}\vec{\tau }\;dt\right|}{\int_{0}^{T}|\vec{\tau }|dt}\right]$$

The OSI has a range from 0 to 0.5. Positions at the wall that experience flow in one consistent direction have an OSI of 0. An OSI of 0.5 is indicative of flow in two opposite directions alternating equal amounts of time during a cycle. TAWSS and OSI showed to provide complementary information [34]. TAWSS is indicative for the average WSS magnitude throughout a cardiac cycle, while OSI identifies regions exposed to oscillatory flow regardless of the magnitude of WSS. Both TAWSS and OSI were designed to identify regions at risk of developing plaque [35]

Previous investigations have established a cut-off of 0.5 Pa for low WSS. Areas exposed to WSS below this threshold can be regarded to be at risk. To resulting areas of low WSS in the phantoms (lWSS_phantom_) and in the reconstructions from the readers (lWSS_reader_) were compared using the similarity index (SI)[31]. This index determines the overlap of the areas and is defined as:

$$SI=\;\frac{2\left({lWSS\;}_{phantom}\;\cap \;{lWSS\;}_{reader}\right)}{{lWSS\;}_{phantom}+\;{lWSS\;}_{reader}}$$

The SI ranges between 0 and 1, where 0 indicates no overlap and 1 indicates full overlap. The SI values for the steady-state computations were presented. Because the areas exposed to TAWSS below 0.5 Pa were small, minor deviation could quickly lead to misrepresentative SI values.

**Fig 2:**
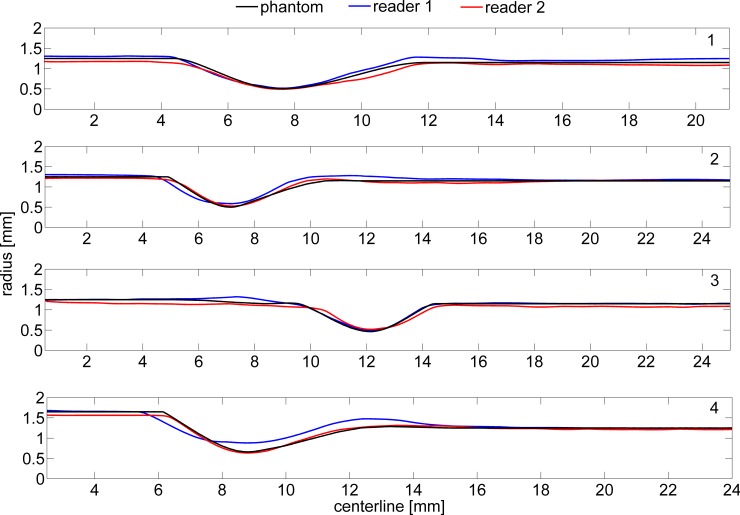
The radii of the main branch of the four original phantom models and of the two reconstructions based on the segmentation by the two readers.

**Fig 3:**
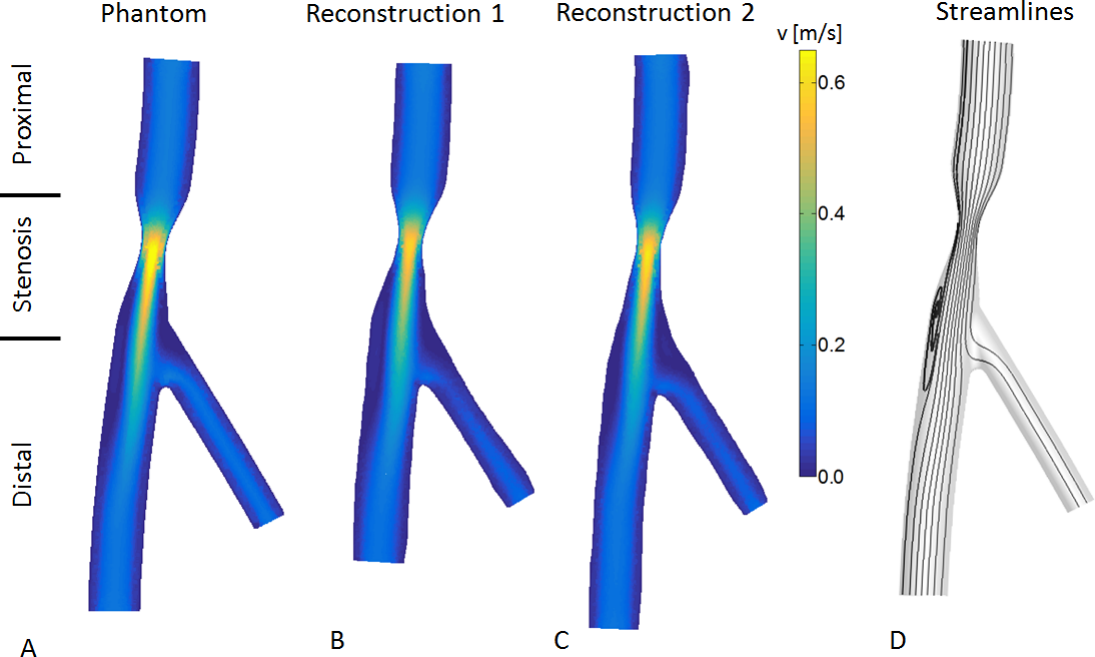
The velocity results of the CFD computations in a plane through the center of phantom 1 and its two reconstructions. In all models a jet forms at the neck of the stenosis and directly distal of the stenosis a recirculation zone is observed. **A:** Results in the phantom model. **B:** Reconstruction based on the segmentations from reader 1. **C:** Reconstruction based on the segmentations from reader 2. **D:** General streamlines observed in the computations.

A second quantitative analysis was comparing the axial and circumferential means per region of the bifurcations. Three regions were investigated separately: the proximal region, the stenosis region and the distal region. The proximal and distal regions were defined as the region where the diameter of the phantom was constant (Figs 2 and 3). Wentzel et al. showed that relevant information can be extracted from the axial mean at 22.5° intervals [36]. Stone et al. showed that taking the full circumferential mean is useful to derive clinically relevant information from WSS computations [4]. The 3D WSS maps were converted to a 2D coordinate system to allow for a direct quantitative comparison. First, planes were defined perpendicular to the centerline at 0.45 mm intervals. Those planes were subdivided in 22.5° intervals. The average WSS values within the resulting bins were mapped to a 2D representation. The 2D maps from the phantom and two readers were clipped to represent identical areas.

### 2.6 Statistics

In order to test if two methods provided equal outcomes, a noninferiority test was employed [37]. A significance level of α = 0.05 rejects the null hypothesis which states that the absolute WSS difference between the phantom and the reader is larger than a chosen margin. In this study that margin was based on the resolution limitations of angiography and the effect this has on the precision with which WSS can be computed. The WSS is defined as the derivative of the velocity normal to the wall. With a definition for the velocity profiles in the proximal region given by Schrauwen et al., an analytical expression for the WSS was derived [38]:

$$\tau =\frac{q\left(\beta +2\right)\mu }{\pi a^{3}}$$

Here μ was viscosity and the wall was defined at r = a. Parameter β describes the shape of the velocity profile and q is flow. Due to the resolution limitation of angiography there is an uncertainty δ on the wall reconstruction:

$$a^{\prime }=a\pm \delta$$

This directly affects the computed WSS:

$$\tau^{\prime }=\tau (a^{\prime })$$

The relative error ($\hat{\varepsilon }$) between the actual WSS and the WSS computed using the reconstruction therefore becomes:

$$\hat{\varepsilon }=\left(1-\frac{\tau^{\prime }}{\tau }\right)*\;100\%$$

In a coronary artery with a radius of for example 1.25 mm and an angiography system with a resolution of 0.1 mm, the relative error ranges from -28% to 21%. From the analytical solution we know that in the proximal and distal regions the WSS is in the order of 1 Pa. With a conservative assumption of ±25% for the relative error, the equivalence margin was set at 0.25 Pa.

Due to more complex definition of OSI it is challenging to define an equivalence margin. Nonetheless, the maximum in the axial and circumferential means of the OSI were 0.2. With the ±25% relative error in mind, the equivalence margin for OSI was set at 0.05. This value is in accordance with a previous study stating that an OSI of 0.05 is not considered physiological significant [39].

The overall agreement of the WSS was assessed per region. To this end the percentage of area was reported where the computed WSS difference was less than the defined equivalence margin.

## Results

Fig 2 shows the radius in the phantom and two readers of the four phantoms. For both readers the mean absolute difference (<δr>) between the radius of the phantom and the radius of the reconstructions was less than 0.10 mm (Table 1). Only the reconstruction by reader 1 of the stenosis in phantom 4 had a difference of 0.21 mm. In 7 out of 8 the mean Hausdorff distance was within 0.20 mm, and in only one reconstruction this was 0.23 mm. The flow rates in the simulation were based on an inlet peak velocity of 15cm/s. The inflow is acquired by multiplying the velocity with the inlet area. The deviations in the reconstruction of the inlet area resulted in different inflow rates.

**Table 1:** Characteristics of the reconstructions of the four phantoms based on the segmentations of two readers. The mean absolute difference (<δr>) between the radius of the phantom and the radius of the reconstruction is analyzed in the proximal, stenosis and distal region.

	<δr> prox [mm]	<δr> sten [mm]	<δr> dist [mm]	Length stenosis [mm]	Mean Hausdorf distance [mm]	Area Stenosis [%]	Inflow [ml/s]	outflow ratio [%/%]
**Phantom 1**								
**AD**				7,5		60.0	0.37	25 / 75
**Reader 1**	0.05±0.00	0.01±0.01	0.08±0.03	7,5	0.14	60.4	0.40	25 / 75
**Reader 2**	-0.08±0.00	-0.01±0.00	-0.04±0.02	7,9	0.14	58.0	0.32	20 / 80
**Phantom 2**								
**CAD**				7,0		60.0	0.37	24 / 76
**Reader 1**	0.03±0.04	0.07±0.01	0.05±0.03	6,4	0.16	54.1	0.38	23 / 77
**Reader 2**	-0.04±0.01	0.02±0.02	-0.02±0.02	5,8	0.12	56.5	0.34	22 / 78
**Phantom 3**								
**CAD**				5,0		60.0	0.37	24 / 76
**Reader 1**	0.01±0.01	0.03±0.01	0.01±0.01	5,1	0.20	61.0	0.35	23 / 77
**Reader 2**	-0.08±0.02	0.05±0.01	-0.07±0.01	4,8	0.10	56.6	0.33	24 / 76
**Phantom 4**								
**CAD**				6,5		60.0	0.64	48 / 52
**Reader 1**	0.02±0.01	0.21±0.01	0.10±0.34	7,0	0.23	47.2	0.64	48 / 52
**Reader 2**	-0.08±0.00	-0.03±0.01	0.03±0.12	6,5	0.12	59.4	0.58	45 / 55

### 3.1 Velocity

The flow rates in the simulation were based on an inlet peak velocity of 15cm/s. By multiplying the velocity with the inlet area the inflow is acquired. The deviations in the reconstruction of the inlet area resulted in different inflow rates. Fig 3A–3C shows the computed velocity fields of the steady-state computations in a plane through the center of phantom 1 and the two reconstructions as an example. In all three reconstructions a jet formed at the stenosis, with a peak velocity of 0.69 m/s, 0.59 m/s and 0.63 m/s in the phantom and two reconstructions respectively. Fig 3D illustrates the streamlines in these geometries. Downstream of the stenosis a recirculation zone was formed and the main direction of the jet was shifted towards the opposite wall. The deviations observed in the reconstruction of the stenosis region, caused alterations in the length and width of the recirculation zone. The transient computations showed a similar velocity field. The recirculation zone was present during the whole cycle, but differed in size due to the pulsation.

### 3.2 Wall shear stress

**Fig 4:**
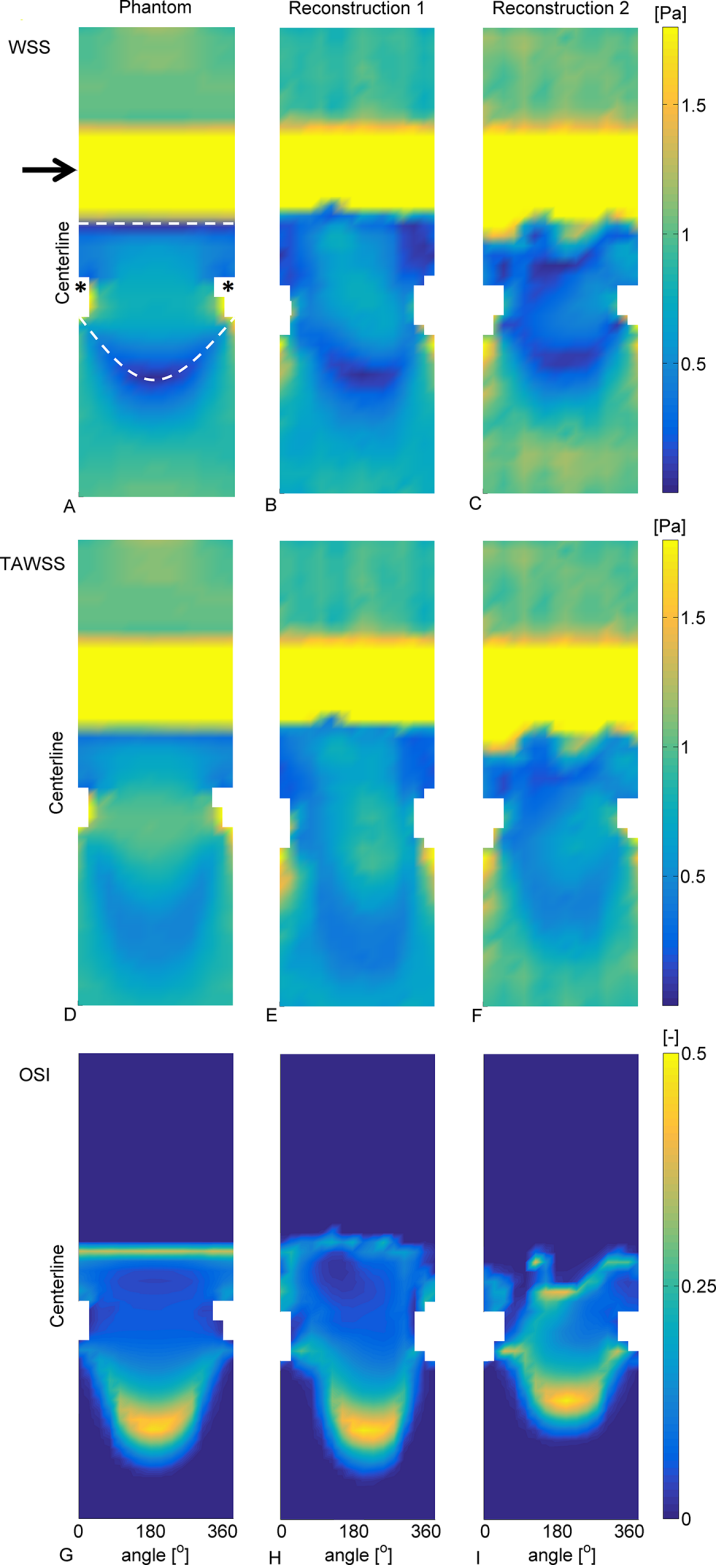
2D maps of the WSS, TAWSS and OSI in the main branch phantom 1. The arrow indicates the stenosis and the asterisk the location of the side branch. **A-C:** Results of the WSS in the phantom (A), reconstruction 1 (B) and reconstruction 2 (C). The color map was saturated to visualize the low WSS. In the area directly distal to the stenosis there was retrograde flow (in between the white dashed lines). The crescent-shaped line where the WSS is zero indicates the transition from retrograde to antegrade flow. **D-F:** TAWSS results. Similar patterns were observed as for the WSS, although for the TAWSS the results were more averaged out. **G-I:** OSI results. Due to the pulsatility a zone of high oscillatory shear is formed in the distal region. This zone is indicative for the movement of the transition zone from retrograde to antegrade flow throughout a cycle.

2D representations of the 3D WSS results in the main branch of phantom 1 are given in Fig 4. The color maps are saturated to visualize the low WSS patterns. The white areas correspond to the location of the side branches (asterisk). Fig 4A shows the WSS magnitude from the steady-state computations in the phantom. In the proximal region the WSS was fairly constant. Distal of the stenosis a crescent-shaped area of low WSS occurs, caused by the recirculation zone. The area between the white dashed lines had retrograde flow near the wall. The point where the WSS is zero indicates the transition to antegrade flow downstream of the crescent-shaped area. The differences in the low WSS patterns between the phantom and the reconstructions are related to the differences seen in the jet formation and the resulting recirculation zone (Fig 4B and 4C). The area of low WSS in reconstructions 1 was skewed caused by a slight offset in the angle of the side branch, and in reconstruction 2 this was smaller. Directly distal of the stenosis in reconstructions 1 and 2 the transition to low WSS shows a ridged edge caused by unevenness in the reconstructions. The TAWSS had the same pattern as the WSS, although the mean TAWSS and its gradients were lower due to the pulsation of the flow (Fig 4D–4F) The pulsation also resulted in a point in the distal region subjected to high oscillatory flow, having an OSI of 0.5 (Fig 4G–4I). The crescent-shaped zones of elevated OSI corresponded to the zones of low WSS and low TAWSS.

As a quantitative comparison the areas exposed to low WSS in the phantoms and the reconstructions were compared. In the steady computations on average 11.5% of the phantom was exposed to low WSS, in reader 1 this was 14.8% and in reader 2 this was 12.8%. Hence, the mean difference of area exposed to low WSS was 2.8 percentage points. In the transient computations on average 6.8% of the phantom was exposed to low TAWSS, this was 13.3% for reader 1 and 10.0% for reader 2. For the transient computations the mean difference was 5.1 percentage points.

**Fig 5:**
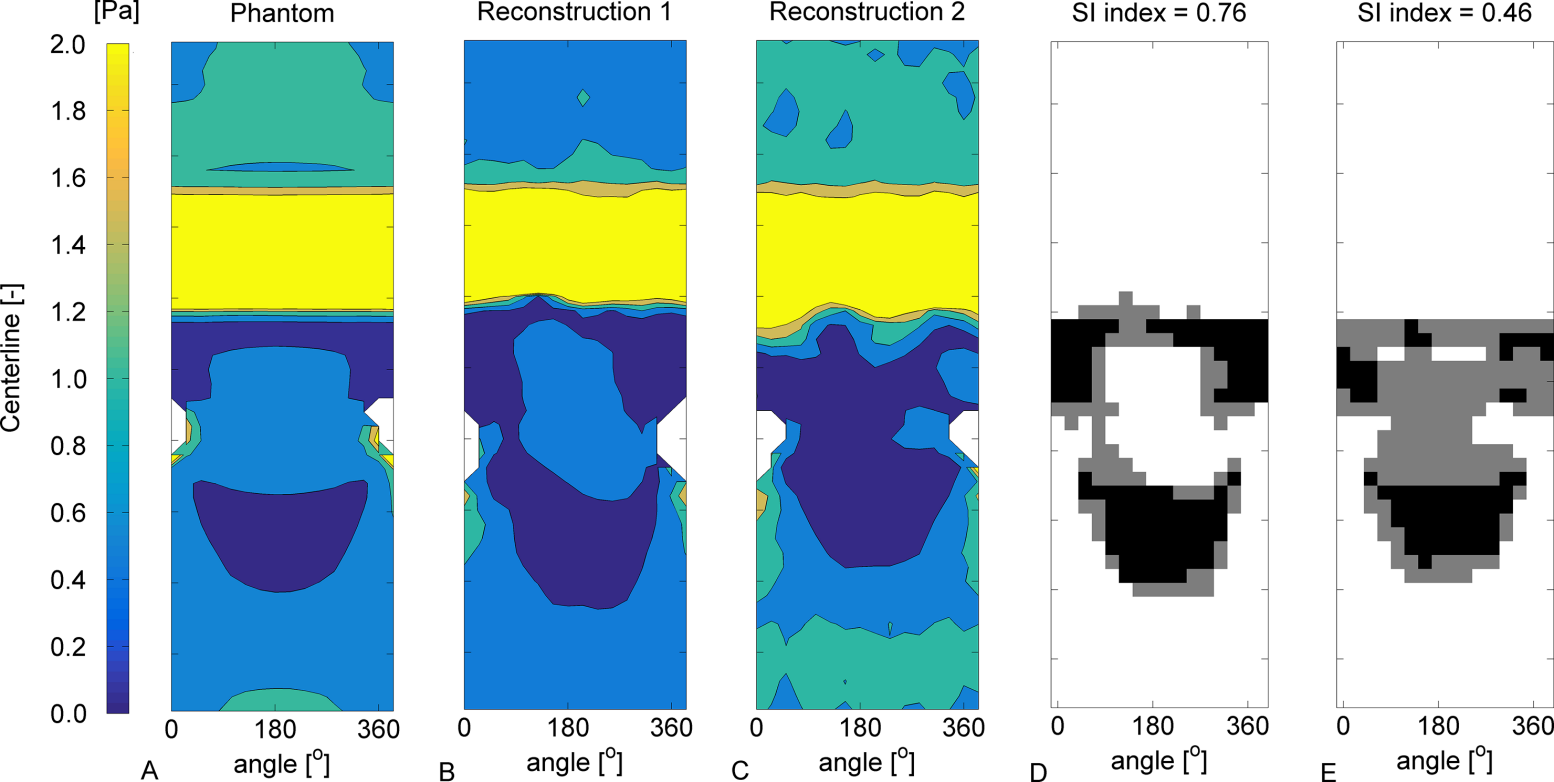
Analysis of SI of WSS in phantom 1 and the corresponding reconstructions. A: Contour plot of the WSS. Similar as Fig 4 a low WSS region is located directly distal of the stenosis and distal of the side branch. B: Contour plot of the WSS in the reconstruction based on the segmentation from reader 1. C: Contour plot in reconstruction 2. D: Plot of the SI between the phantom and reconstruction 1. The white indicates WSS above 0.5 Pa in both the phantom and the reconstruction. Gray indicates low WSS in either the phantom or the reconstruction. Black indicates low WSS in both the phantom and the reconstruction. E: SI between the phantom and reconstruction 2.

In Fig 5 the SI of WSS is analyzed for phantom 1.Panels A-C show contour plots of the WSS in phantom and the 2 reconstructions. The crescent shaped areas of low WSS in the distal regions qualitatively match, but the location of the low WSS is influenced by the reconstruction errors. In Fig 5 panels D and E give a visualization of the SI. The white areas indicate where the WSS was above 0.5 in both the phantom and one of the reconstructions, and the black areas indicates where the WSS was lower than 0.5. The gray areas in panels D and E indicate low WSS in only found in either the phantom or the reconstruction. The SI for the reconstruction 1 was 0.76 and for reconstruction 2 this was 0.46. In Table 2 all SI values are given. In phantom 1 and 3 a relatively high SI was found for the steady-state computations. In phantom 2 the SI was reasonable and in phantom 4 it was low.

**Table 2:** SI of the areas of the phantoms and the reconstructions exposed to low WSS.

	Similarity index	
Reader	1	2
**Phantom 1**	0,76	0,46
**Phantom 2**	0,40	0,36
**Phantom 3**	0,75	0,37
**Phantom 4**	0,03	0,52

The mean absolute differences in WSS between the four phantoms and their reconstructions were computed. The mean absolute WSS difference in the proximal regions ranged from 0.05±0.04 Pa to 0.17±0.06 Pa. In the stenosis region this ranged from 0.73±1.14 Pa to 2.59±4.00 Pa, and in the distal region from 0.12±0.15 Pa to 0.36±0.42 Pa. This lead to average absolute difference of the two readers in the four phantoms of 0.08 Pa in the proximal region, 1.34 Pa in the stenosis region and 0.20 Pa in the distal region. The TAWSS resulted in similar differences; on average the differences in the proximal, stenosis and the distal region were 0.09 Pa, 1.59 Pa and 0.23 Pa respectively. The OSI in the proximal region was 0 and no differences were observed. On average the mean absolute difference of the OSI in the stenosis region was 0.03, and in the distal region this was 0.05.

### 3.3 Averaging and equivalence test

**Fig 6:**
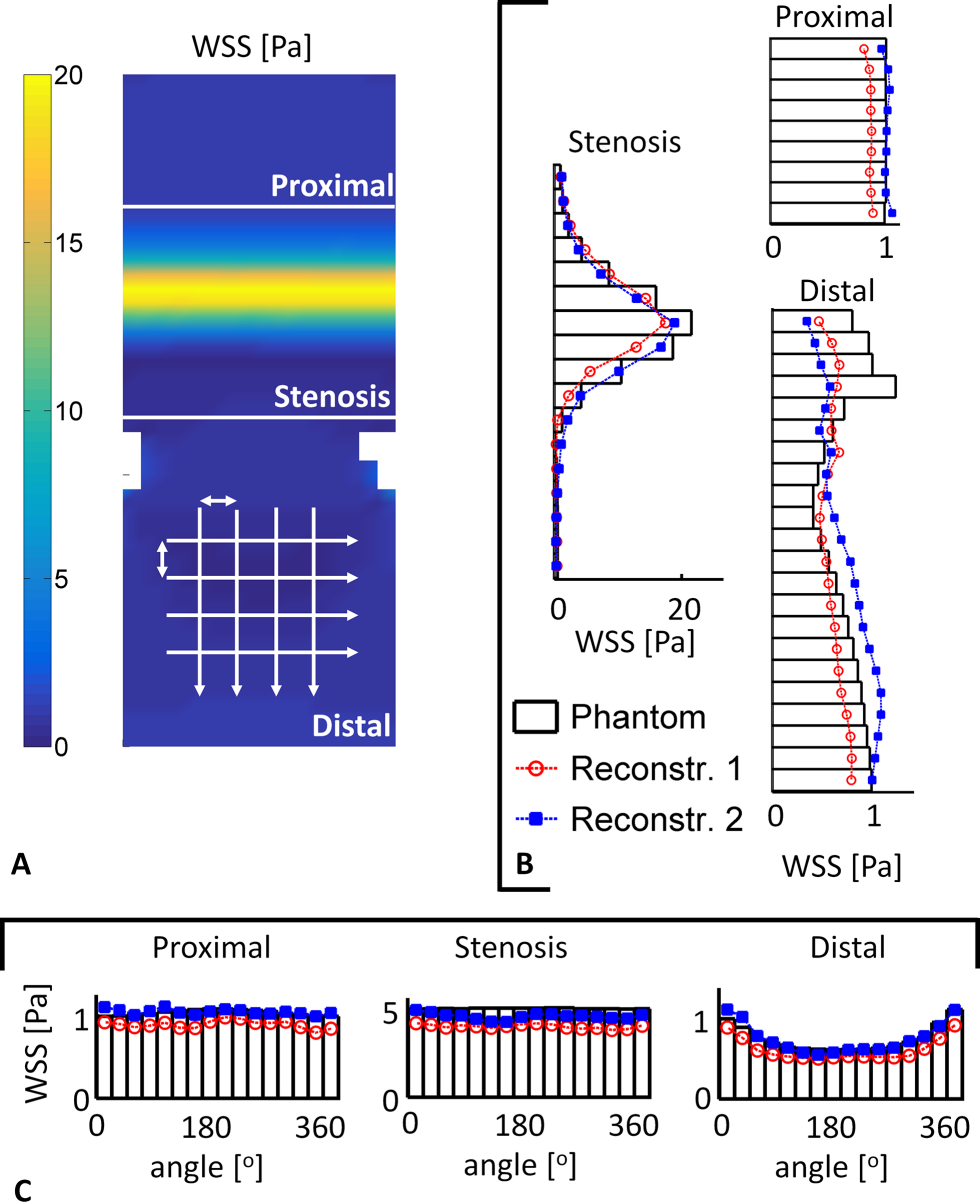
Quantitative analysis of the WSS in phantom 1. The white lines mark the three regions. **A:** 2D WSS map of phantom 1.The arrows indicate the direction of the averaging procedure. **B:** The circumferential means in the three regions. The bars indicate the mean circumferential WSS in the phantom and the lines the WSS from reconstruction 1 (red circle) and reconstruction 2 (blue square). In the proximal and stenosis region the WSS from the readers match the WSS in the phantom. In the distal region more pronounced differences are observed, primarily directly after the stenosis. **C:** Axial means in the three regions. In all regions the WSS from the readers match the WSS in the phantom.

**Fig 7:**
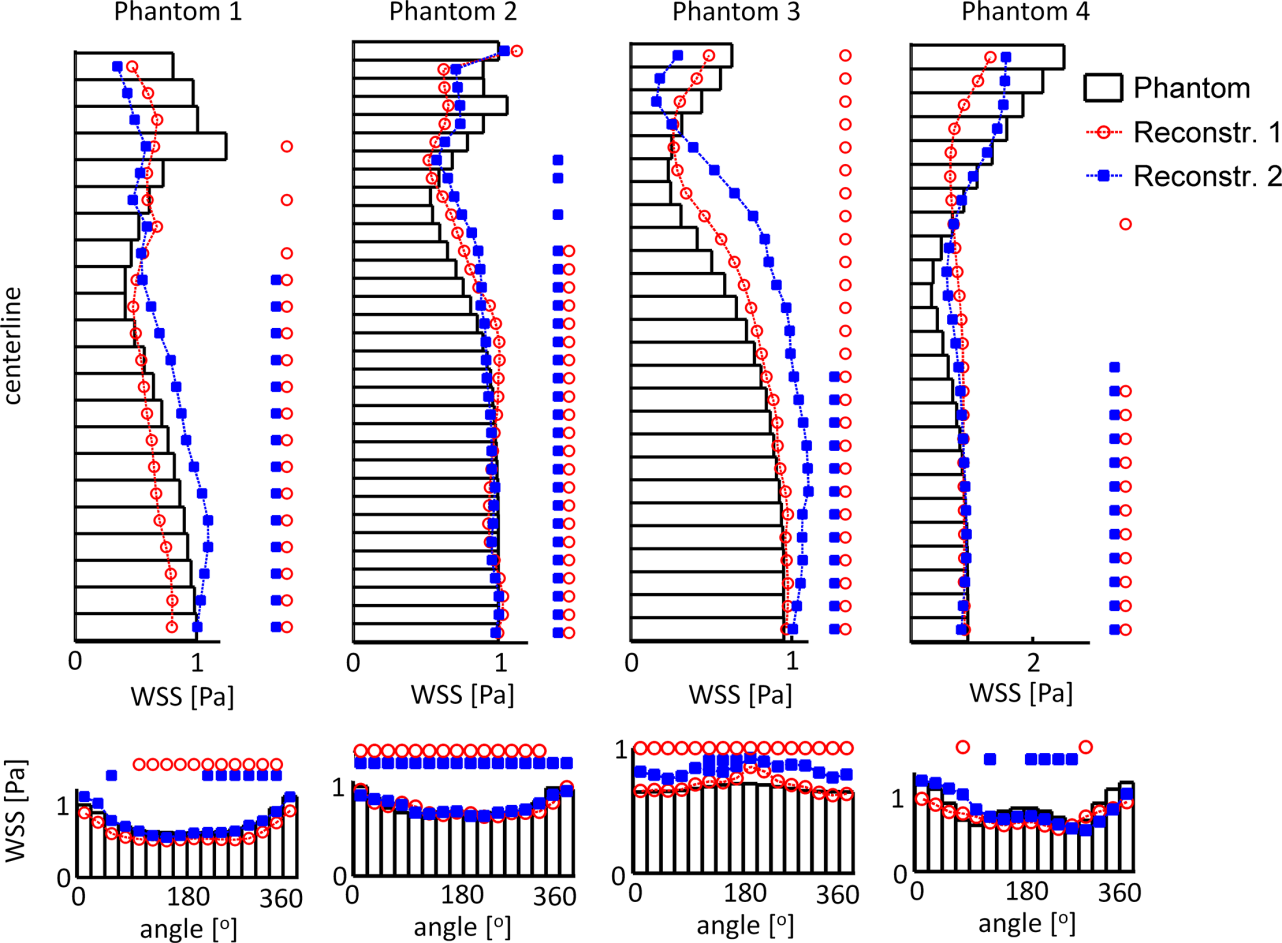
Circumferential and axial mean WSS in the four phantoms and reconstructions 1 and 2. The asterisks indicate the locations were the WSS in the phantom and the readers did not reach clinical equivalence. Particularly in the region directly distal of the stenosis the WSS is not clinically equivalent. Far distal the circumferential means are clinically equivalent. The axial means in the readers are similar as the phantom, though equivalence in not reached everywhere.

Fig 6 shows the axial and circumferential means of phantom 1. The axial means in all regions of the reconstructions matched those computed in the phantom, especially immediately distal of the stenosis. The circumferential means in the proximal and stenosis region agreed nicely, although it was consistently lower in reconstruction 1. In the distal region the differences between the reconstructions and the phantom became more pronounced. This was consistent in all four phantoms (Fig 7). The symbols in Fig 7 indicate where the results were equivalent. Particularly immediately downstream of the stenoses the computed WSS were not equivalent.

In Table 3 the average percentage of equivalence per region is reported of all four phantoms. In the proximal regions the means in both directions were clinically equivalent. In the stenosis region, where the WSS were much higher, only the circumferential means shows some overlap in the order of approximately 30%. In the distal region the percentage of equivalence ranges from 40% to 80%. For the TAWSS, the percentage of equivalence in the regions was similar as reported for the WSS. The OSI in the proximal regions was 0 in all cases and therefore tested equivalent. The circumferential means in the stenosis region had more than 80% equivalence in six cases. In the distal region the equivalence typically ranged from 40% to 80% in both directions.

**Table 3:** Overview of the average percentage of area where equivalence was observed in the four phantoms for the WSS, TAWSS and OSI.

**Circumferential**	**Percentage of equivalence**		
	**proximal**	**stenosis**	**distal**
**Steady**	100	18	66
**TAWSS**	100	20	58
**OSI**	100	81	49
**Axial**	**Percentage of equivalence**		
	**proximal**	**stenosis**	**distal**
**Steady**	99	0	58
**TAWSS**	100	0	53
**OSI**	100	64	45

### 3.4 Wall shear stress vs. time-averaged wall shear stress

The middle row of Fig 4 shows that qualitatively the TAWSS was similar to the WSS, although the low WSS in the distal region was more spread out. The equivalence test on the results from the phantom computations revealed that the proximal region was entirely equivalent (Table 4). The circular means in the stenosis region were equivalent in 70% of that region. In the distal region the equivalence in both circular and axial direction ranged from 50% to 80%.

**Table 4:** Percentage of equivalence between the WSS and TAWSS in the two original phantoms.

**Circ**	**proximal**	**stenosis**	**distal**
1	100	71	68
2	100	73	70
3	100	67	77
4	100	74	56
**Axial**	**proximal**	**stenosis**	**distal**
1	100	0	50
2	100	0	81
3	100	0	63
4	100	0	63

## Discussion

This study investigated the influence of angiography-based reconstructions on velocity and WSS computations in models of diseased coronary bifurcations. WSS computations in four phantom models served as the ground truth and were compared to the outcome of WSS computations in angiography-based reconstructions. Proximal to, and at the stenosis the WSS results quantitatively agreed nicely. Distal to the stenosis the same phenomena were observed, but subtle deviations in the reconstruction caused differences in jet characteristics and therefore the size and location of the low WSS regions. An equivalence test revealed that the results in the proximal part were statistically equal. However, directly distal of the stenosis significant variations were observed between the WSS in the phantoms and the reconstructions.

### 4.1 Velocity

Several studies focusing on the accuracy of the 3D reconstructions previously validated the CAAS 3D software. The most recent study reported an overall measurement error of approximately 0.02 mm for the lumen and 0.04 mm for the stenosis diameter [17], similar to the findings in this study. The previously reported Hausdorff distances between ground truth and reconstructed scaled up coronary artery geometries are also comparable [27]. Although the segmentation errors were small, they directly influenced the velocity patterns. Deviations in the reconstruction of the stenosis were immediately reflected in the peak velocity of the jet. The peak velocity in turn influenced the size and shape of the recirculation zone. The segmentation errors downstream of the stenosis mainly influenced the orientation of the jet.

The reason that this jet could be influenced by the small reconstruction errors is related to design of the phantom. The planar phantom models with concentric stenoses were designed to investigate the precision of the image reconstruction techniques with regard to length and stenosis degree. However, this design resulted in symmetry of the flow, what could easily be disturbed by small deviations in the reconstructions. Curvature and eccentricity of stenoses on the other hand, can both cause helical flow patterns [20,39,40]. It was hypothesized that helical flows induces more stability [41–43]. So the computations in this study could be regarded as more challenging and provided a more rigorous test than curved models with eccentric stenoses might have been. For clinical purposes it would be interesting next step investigate this type of models.

### 4.2 Wall shear stress

The WSS in the two reconstructions were similar to the WSS in the phantoms. In the proximal and far distal regions the WSS agreed particularly well, and the WSS values were equivalent. From these results it follows that WSS can accurately be computed with angiography-based 3D reconstructions in coronary arteries suffering from early stage atherosclerosis. The accurate prediction of absolute high and low WSS regions at and distal of the–admittedly challenging- symmetric stenoses proved to be more difficult. At the neck of the stenosis the maximal WSS differed between the phantom and the reconstructions. In these high WSS regions, reconstruction errors significantly influenced the absolute values, making studies focusing on absolute WSS for rupture risk prediction based on angiography alone difficult [35]. The regions directly distal of the stenoses were all exposed to low WSS, but the size of those regions varied. This was reflected in the results from the similarity index and the equivalence test. In the presence of a more advanced stenosis, angiography-based reconstruction might not suffice in providing the required precision to assess low WSS immediately distal of a–concentric- stenosis. An accurate WSS assessment in this region is important because of possible plaque progression [35]. To obtain the required reconstruction precision, intra-vascular imaging techniques, such as IVUS or OCT, can be used [12,15,44,45]. If angiography alone will be used, concentric stenoses must be regarded with caution. Furthermore, normalized WSS has proven to be a more robust parameter than absolute WSS [46], and might be a useful alternative to study the relationship between WSS and atherosclerosis in general.

Similar to previous reports [20,39,45], the results from steady-state and transient simulations matched qualitatively. With the noninferiority test this study demonstrated to what extent these results were statistical equivalent. In the proximal and far distal region they were equivalent, but mainly in the area directly distal of the stenosis the results differed. Thus, steady-state simulations might be valuable in mildly diseased coronary arteries, but the low WSS are not captured well in case of a lumen intruding plaques. Future studies should be directed to find evidence that links WSS from steady-state computations to the natural history of atherosclerosis.

As stated by Peiffer et al., a point-to-point analysis of WSS in coronary arteries represents an ideal method for determining relationships between WSS and wall parameters [47]. Since WSS is a derived value from the velocity field at the wall it is highly sensitive to deviations in the reconstruction of the lumen. Even in the proximal region, where the WSS results matched to a high degree, a point-to-point comparison would still render statistically different. Numerous studies therefore employed other averaging techniques to analyze WSS. Goubergits et al. compared WSS in a phantom and an angiography-based reconstruction, and performed an analysis by construction normalized histograms [19]. This allowed for an elegant comparison of the global WSS distribution, but local information is lost. Other investigators used either axial or circumferential averaging [4,36]. While this is a form of data reduction as well, some local information is retained. Both averaging techniques were used in this study, and subsequently a test was introduced to find statistical evidence for equivalence of the computed WSS. The combination of averaging techniques and testing for statistical equivalence could be a powerful tool in longitudinal studies that investigate the role of WSS in atherosclerotic disease progression.

## Limitations

This study was limited at some points. First, in this study phantom models were used, while the final goal is an application in human coronary arteries. The main advantage of using phantoms is that the exact geometry was known allowing for a quantitative comparison of WSS patterns. Also by using these models motion artifacts were excluded. This allowed ruling out all effects other than reconstruction errors influencing WSS computations. However, in a clinical setting motion artifacts might play an important role, even when applying high frame rate imaging. For example, in diastole the velocity of a coronary artery can be in the order of 5 mm/s due to cardiac motion [48]. With a frame rate of 30 frames per second the resulting error can be in the same order as the imaging resolution. Future studies should focus on finding the influence of motion artifacts in angiography-based 3D reconstructions.

Next, in this study the phantoms were filled with contrast agent. In a clinical setting the imaging quality could be influenced by the mixing of the contrast agent with the blood. Similarly, the presence of bone and other tissue of the thorax that surround coronary arteries could influence the quality as well.

Finally, the outcome of the noninferiority test critically depends on the chosen margin of equivalence [37]. Our study demonstrated the added value of this technique, but also revealed the difficulty in setting the margins. Future studies should strive to reach a consensus on what margin can be regarded as clinically equivalent. Knowing that the precision of WSS computations cannot excel beyond the resolution limits of the used imaging modality, it seems compelling to take into account the sensitivity of the error due to the resolution limitation.

## Conclusion

In conclusion, this study showed that WSS can accurately be computed within angiography-based 3D reconstructions of coronary arteries with early stage atherosclerosis. Fusion of angiography with intra-vascular techniques, such as IVUS or OCT, might be of added value for WSS computations in the presence of more advanced atherosclerosis.
